# Rapid detection of dermatophytes from skin and hair

**DOI:** 10.1186/1756-0500-2-60

**Published:** 2009-04-18

**Authors:** Jaya Garg, Ragini Tilak, Atul Garg, Pradyot Prakash, Anil Kumar Gulati, Gopal Nath

**Affiliations:** 1 Department of Microbiology, Institute of Medical Sciences, Banaras Hindu University, Varanasi, India

## Abstract

**Background:**

Dermatophytes are a group of closely related keratinophilic fungi that can invade keratinized humans and animals tissues such as skin, hair and nails causing dermatophytosis. They are an important cause of superficial fungal infection.

**Findings:**

Conventional methods like potassium hydroxide (KOH) microscopy and fungal culture lacks the ability to make an early and specific diagnosis. In this study we have evaluated nested Polymerase chain reaction (PCR) using primers targeting dermatophyte specific sequence of chitin synthase 1 (*CHS1*) gene and compared with conventional test. A total of 155 patients clinically suspected with dermatophytosis were included in the study. Of which 105 specimens were skin scrapings and 50 were hair. KOH microscopy, fungal culture and first round and nested PCR were done on clinical specimens, and results compared. Nested PCR for dermatophytes was positive in 83.8% specimens, followed by KOH microscopy (70%), first round PCR (50.8) and fungal culture (25.8).

**Conclusion:**

Results indicate that nested PCR may be considered as gold standard for the diagnosis of dermatophytosis and can aid the clinician in initiating prompt and appropriate antifungal therapy.

## Introduction

Superficial fungal infections are common skin diseases, affecting millions of people worldwide [1]. These infections occur in both healthy and immunocompromised patients and etiologic agents consist of dermatophytes, yeasts and nondermatophyte molds. Dermatophytes are responsible for most superficial fungal infections [2] and the estimated lifetime risk of acquiring a dermatophyte infection is between 10–20%. [3]

Dermatophytes are a group of closely related keratinophilic fungi that can invade keratinised humans and animals tissues such as skin, hair and nails causing dermatophytosis.[4] Dermatophytes consist of three genera *Trichophyton*, *Microsporum*, and *Epidermophyton *[5]. Worldwide the most common cause of tinea pedis, tinea unguium (onychomycosis), tinea cruris, tinea mannum, tinea corporis, and tinea faciei is *Trichophyton rubrum *[6]. Other frequently implicated agents include *Trichophyton mentagrophytes*, *Microsporum canis*, *Microsporum gypseum *and *Epidermophyton floccosum *[7]. The laboratory diagnosis of dermatophytosis routinely involves direct microscopic examination of clinical specimen followed by invitro culture techniques. Microscopic identification of fungal elements directly from clinical specimen is a rapid diagnostic method but it lacks specificity and sensitivity, with false negative results in up to 15% cases [8]. In vitro culture is a specific diagnostic test but it is slow technique, and may take up to 8 weeks to give the results [9].

The advent of molecular technology has enabled the development of techniques like polymerase chain reaction, which is a highly sensitive and specific test and can be used for diagnosis of various microorganisms including fungal pathogens. In our previous study we have evaluated nested PCR targeting the Chitin Synthase 1 (*CHS1*) gene (DDBJ accession no.-AB003558) shared by three genera, i.e., *Trichophyton*, *Epidermophyton*, and *Microsporum*, in patients with clinically suspected cases of onychomycosis [10]. In this study we have evaluated a nested PCR targeting the *CHS1 *gene in skin and hair specimen of patients clinically suspected with dermatophytosis.

## Materials and methods

A total of 155 patients clinically suspected with dermatophytosis were included in the study irrespective of their age or gender. The most common clinical presentation among skin dermatophytosis (n = 105) was tinea pedis (*n *= 51), followed by tinea corporis (*n *= 19), tinea cruris (*n *= 20), and tinea manuum (*n *= 15). Among hair dermatophytosis (n = 50), tinea capitis including kerion and black dot (n = 26) was most frequent followed by tinea barbae (n = 24). The study participants included some patients (n = 87) who had not clinically responded to 2 to 3 months of empirical oral antifungal treatment.

In skin dermatophytoses the clinical specimens collected were epidermal scales. The scales were scrapped from near the advancing edges of the lesions after disinfecting the lesions with 70% alcohol. Where the advancing edges were not evident, scrapings were collected from areas representing the whole infected area. In hair dermatophytoses basal root portion of hair was collected by plucking the hair with sterile forceps. In cases with black dot, scalpel was used to scrape the scales and excavate small portions of the hair roots.

The collected specimens were divided into three portions. The first portion of the specimens was examined microscopically using 20% potassium hydroxide (KOH) with 40% dimethyl sulfoxide. The second portion was cultured on Sabouraud's dextrose agar containing chloramphenicol (0.05%) with and without cycloheximide (0.5%) and incubated at 25°C for 4 to 6 weeks. Clinical isolates were identified on the basis of phenotypic characteristics of the colonies, microscopic examination of lactophenol cotton blue wet mounts, and physiological tests such as urease production, in vitro hair perforation, and nutritional requirement tests.

DNA extraction was performed on the third portion of specimen by crushing them in liquid nitrogen. The crushed specimen were suspended in 200 μl of Tris-EDTA buffer and subjected to repeated freezing and thawing. Then, 300 μl of 0.1% Triton X-100 (pH 8) and 2 μl of proteinase K solution (20 mg/ml) were added and incubated for 2 h at 65°C. The extracted DNA was purified by the phenol-chloroform- isoamyl alcohol (25:24:1) method and resuspended in 30 μl of Tris-EDTA buffer. First-round PCR was performed using primer pairs CHS1 1S (5'-CAT CGA GTA CAT GTG CTC GC-3'; nucleotides [nt] 70 to 89) and CHS1 1R (5'-CTC GAG GTC AAA AGC ACG CC-3'; nt 485 to 504). These primers amplify a 435-bp DNA fragment of the dermatophyte-specific *CHS1 *gene sequence of *Arthroderma benhaemiae*, a teleomorph of *Trichophyton mentagrophytes *(DDBJ accession no. AB003558). Nested PCR was performed by designing a novel set of primers, JF2 (5'-GCA AAG AAG CCT GGA AGA AG-3'; nt 111 to 130) and JR2 (5'-GGA GAC CAT CTG TGA GAG TTG-3'; nt 378 to 398), amplifying a DNA fragment of 288 bp from the internal sequence of the amplicon obtained from first-round PCR.

The PCR mixture (25 μl) for first-round PCR contained 2.5 μl of 10× buffer (100 mM Tris-HCl, 500 mM KCl, and 0.8% [vol/vol] Nonidet P40; MBI Fermentas, Hanover, MD), 1.1 μl of (1.5 mM) MgCl_2 _(MBI Fermentas), 25 pmol each of primers CHS1 1S and CHS1 1R (Operon, Cologne, Germany), 1 μl of deoxynucleoside triphosphate mix (MBI Fermentas), 1 U of *Taq *DNA polymerase (MBI Fermentas), and 15 μl of DNA template. Deionised water was added subsequently to achieve the reaction volume. The reaction mixture was initially denatured at 94°C for 3 min, followed by 30 cycles of denaturation at 94°C for 60 s, annealing at 60°C for 75 s, and extension at 72°C for 120 s. This was followed by a final extension step for 7 min at 72°C in a thermal cycler (Biometra, Goettingen, Germany). The PCR mixture for nested PCR consisted of 25 pmol of primers JF2 and JR2 along with a 1:6 diluted product of the primary cycle as the DNA template; the rest of the constituents were the same as those described above. The running conditions of nested PCR were similar to the first-round PCR except that an annealing temperature of 63°C and 40 cycles were used. Triple-distilled water and DNA of *Trichophyton mentagrophytes *were used as the negative and positive controls, respectively.

**Figure 1:**
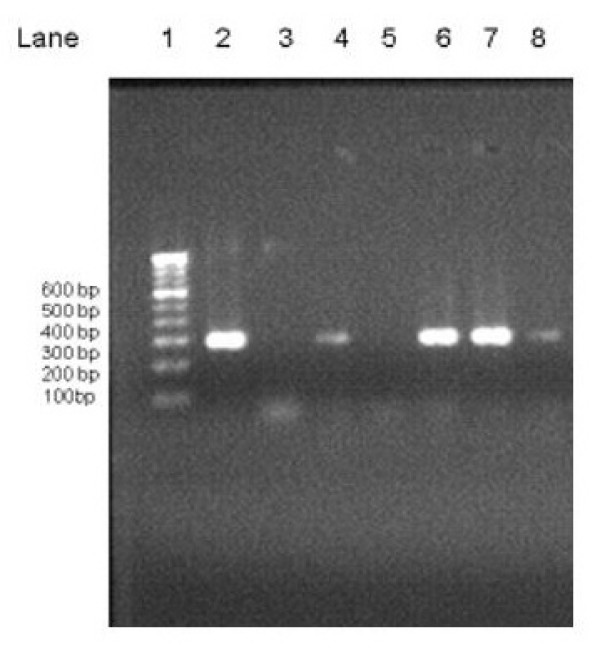
**Results of Nested PCR of clinical specimens of dermatomycosis**. Lane 1, 100 bp DNA ladder (Molecular Marker); Lane 2, Positive Control (288 bp); Lane 3, Negative Control; Lane 5, Nested PCR Negative Cases; Lane 4, 6, 7, 8, Nested PCR Positive Cases.

To document the amplified products, 5 μl of product from first-round PCR and nested PCR was electrophoresed on a 1.5% agarose gel (containing 1.5 μg/100 ml ethidium bromide) in Tris-borate-EDTA buffer, along with the tracking dye bromophenol blue, initially at 100 V for 5 min and then at 80 V for 60 min. Thereafter, bands were visualized under UV light and amplicon of 288 bp was taken as positive for dermatophytes. (Fig. 1)

For statistical analysis sensitivity, specificity, positive and negative predictive value and the likelihood ratios for a positive test result (LR^+^) and a negative test result (LR^-^) were calculated [11].

## Results

Of the 105 clinically suspected cases of skin dermatophytosis, 63.8% (67/105) were positive for fungal elements by KOH microscopy. Dermatophytes were detected in 82.8% (87/105) of the specimens by nested PCR, 49.5% (52/105) by first round PCR and isolated by culture in 23.8%(25/105) cases. Among the dermatophytes isolated on culture *Trichophyton rubrum *was the commonest isolate (48%, 12/25), followed by *T. mentagrophyte *(40%, 10/25), *Trichophyton tonsurans *(8%, 2/25), and *Trichophyton violaceum *(4%, 1/25). Of 80 specimens negative for dermatophyte isolation by fungal culture, 4 specimens were positive for nondermatophytic molds and 12 specimens for *Candida albicans*. 37 (46.2%) specimens were positive by first round PCR and 59 (73.7%) by nested PCR. Of the 87 nested PCR positive specimens *candida albicans *was cultured from 5 specimens, thus nested PCR detecting cases with hidden mixed infections. Nested PCR was positive in 73.7% (28/38) of the KOH microscopy-negative specimens. In addition, all 59 patients on antifungal therapy were positive by nested PCR (Table 1).

**Table 1:** Status of KOH microscopy, fungal culture, first round PCR and nested PCR in suspected cases with skin fungal infections.

**No of cases**	**Patients on treatment**	**KOH** **microscopy**	**Culture shows growth of dermatophyte**	**First round PCR**	**Nested PCR**
**7**	+	-	-	-	+

**9**	-	+	+	-	+

**15**	-	+	+	+	+

**12**	+	+	-	+	+

**15**	+	+	-	-	+

**20**	+	-	-	+	+

**7^a^**	-	+	-	-	-

**5^a^**	+	+	-	+	+

**4^b^**	-	+	-	-	-

**1**	-	-	+	-	+

**10**	-	-	-	-	-

**105**	**59 (56.2%)**	**67(63.8%)**	**25(23.8%)**	**52 (49.5%)**	**87(82.8%)**

Data in parenthesis indicates percentage; ^a ^Culture positive for *Candida albicans*

^b ^Culture positive for non-dermatophytes.

Among 50 clinically suspected cases of hair dermatophytosis, positivity by nested PCR was highest 86% (n = 43/50) followed by KOH microscopy 58% (n = 29/50), first round PCR 52% (n = 26/50) and fungal culture 30% (n = 15/50). Among the dermatophytes isolated, *Trichophyton tonsurans *was the commonest isolate (66%, 10/15), followed by *Trichophyton violaceum *(13%, 2/15), *Trichophyton verrucosum *(13%, 2/15), and *Trichophyton mentagrophytes *(6.6%, 1/15). Nested PCR was positive for 66.6% (n = 14/21) and 80% (n = 28/35) of the KOH microscopy-negative and culture-negative specimens respectively. Of twenty specimens negative both by KOH microscopy and fungal culture, nested PCR was positive in 13 (65%) specimens. In addition, all 28 patients on antifungal therapy were positive by nested PCR (Table 2).

**Table 2:** Status of KOH microscopy, fungal culture, first round PCR and nested PCR in suspected cases with hair fungal infections.

**No of cases**	**Patients on treatment**	**KOH** **microscopy**	**Culture shows growth of dermatophyte**	**First round PCR**	**Nested PCR**
**3**	+	-	-	-	+

**5**	-	+	+	-	+

**9**	-	+	+	+	+

**7**	+	+	-	+	+

**8**	+	+	-	-	+

**10**	+	-	-	+	+

**1**	-	-	+	-	+

**7**	-	-	-	-	-

**50**	**28 (56%)**	**29(58%)**	**15 (30%)**	**26(52%)**	**43(86%)**

On Statistical analysis, sensitivity and specificity of KOH microscopy for skin scrapings was 66.7% and 47.6%, and for hair sample 67.4% and 100% respectively. The sensitivity and specificity of fungal culture for skin scrapings was 29.7% and 100%, and for hair sample 100% and 34.9% respectively.

## Discussion

Dermatophytes are among the few fungi causing communicable diseases; previously most dermatophyte strains had relatively restricted geographical distribution. However recently, dermatophytosis has become one of the most common human infectious diseases in the world and is cosmopolitan in distribution. Dermatophytosis cannot be easily diagnosed on the basis of clinical manifestations as a number of other conditions mimic the clinical presentation. The differential diagnosis of dermatophytoses includes seborrhoeic dermatitis, atopic dermatitis, contact dermatitis, psoriasis, candidal intertrigo, erythrasma, eczema etc [12]. Further it is more difficult to diagnose dermatophytosis in immunocompromised patients, as clinical presentation is often atypical [13].

It is essential that good laboratory methods are available for rapid and precise identification of the dermatophytes involved, in order to apply appropriate treatment and prevention measures. The conventional methods of fungal detection have their own drawbacks; for e.g. KOH microscopy has low specificity and fungal culture is associated with low sensitivity and takes long time. Further dermatophyte isolates from patients on antifungal treatment generally do not show characteristic morphology on culture, thus further compromising the results of culture isolation [9]. The changing profiles of human dermatophytoses among countries have further necessitated the development of improved diagnostic methods for identification of dermatophytes [9]. Thus newer fungal diagnostic methods are need of the hour as identification of the etiological agent is required not only for accurate diagnosis, but also for post-therapeutic strategies [14,15].

The treatment of dermatophytoses would be most appropriate when the selection of antimicrobial agent is based on the identity of the causative agent. For e.g. griseofulvin is effective only for dermatophytic infections, with no activity against *Candida spp*. and nondermophytic molds. Terbinafine shows fungicidal activity against dermatophytes with a cure rate of 80 to 95% but shows only fungistatic activity against *Candida albicans*. For nondermatophytic molds infections, the role of terbinafine is not well defined and topical amorolfine lacquer may be effective for select patients [16]

Recently, molecular biology-based techniques, such as PCR followed by restriction fragment length polymorphism (RFLP) [17], Real time PCR [18] and multiplex PCR assay [19] have been adapted for detection of dermatophytes from clinical specimen. These molecular methods have a good potential to directly detect dermatophytes in clinical specimens; however these methods are yet to be standardised for routine clinical laboratories. PCR – RFLP is a complexive technique with poor discriminative power to make an easy and specific diagnosis. Real time PCR – appears to be promising but is not practical enough for a large number of laboratories that are either small scale or very tightly budgeted.

Very few studies have compared KOH microscopy and culture with direct PCR of clinical specimens In a case study, Nagao et al. detected *Trichophyton rubrum *by nested PCR targeting internal transcribed spacer gene 1 (*ITS1*) in a patient with trichophytia profunda acuta, which was negative by both KOH microscopy and culture [20]. Yan et al evaluated arbitrary primed PCR with conventional methods in 50 tinea corporis and 58 tinea cruris patients and showed that arbitrary primed PCR is a rapid sensitive and specific detection method for dermatophytes from skin scrapings. [21]. Recently bergman et al, performed a PCR-reverse line blot assay on 819 clinical samples (596 nail, 203 skin and 20 hair) and demonstrated a positive PCR-RLB result in 93.6% of 172 culture-positive and microscopy-positive samples.[22]

In our previous study involving 152 clinically suspected patients with onychomycosis it was established that nested PCR might be considered as gold standard for the diagnosis of onychomycosis, where the etiological agents are dermatophytes.

In the present study, nested PCR for both skin and hair dermatophytoses was observed to be more sensitive for the detection of dermatophytes than culture isolation, KOH microscopy, and single-round PCR. The lower sensitivity of single-round PCR compared to KOH microscopy was circumvented in the present study by the use of nested PCR. Further nested PCR is helpful for the diagnosis of cases with dermatophytoses which were recently treated with antifungal agents and showed uncultivable filaments and also grew as spurious molds which were difficult to identify [23]. Further all 24 cases which were positive both by KOH microscopy and culture, were also positive by nested PCR thus demonstrating that nested PCR did not missed even a single known positive case of dermatophyte infection. Of the 87 specimens positive by nested PCR, *Candida albicans *were cultured from 5 specimens, thus detecting cases with hidden mixed infections that clinically manifests in a single lesion. For an appropriate diagnostic test, desirable values for LR^+ ^and LR^- ^should be ≥ 10 and ≤ 0.1, respectively [11]. By considering nested PCR as the gold standard, the LR^+ ^values of KOH microscopy and culture in skin sample were 1.24 and ∞ respectively, while the LR^- ^values were 0.72 and 0.71, respectively. The LR^+ ^value of KOH microscopy and culture in hair specimens was ∞ for both, while the LR^- ^values were 0.33 and 0.66, respectively.

It may therefore be concluded that nested PCR targeting the *CHS1 *gene may be considered the gold standard for detection of dermatophytes in patients with dermatophytoses and can aid the clinician in initiating prompt and appropriate antifungal therapy. This technique is not only rapid but also simple and cheap in comparison to other molecular methods for detection of dermatophytes.

## Competing interests

The authors declare that they have no competing interests.

## Authors' contributions

JG and AG collected the samples and performed the PCR, GN and PP designed the primers and standardised the DNA extraction procedure, RT performed KOH and fungal culture and AKG provided the logistic support and did the critical evaluation of manuscript.
